# A social-ecological database to advance research on infrastructure development impacts in the Brazilian Amazon

**DOI:** 10.1038/sdata.2016.71

**Published:** 2016-08-30

**Authors:** Joanna M. Tucker Lima, Denis Valle, Evandro Mateus Moretto, Sergio Mantovani Paiva Pulice, Nadia Lucia Zuca, Daniel Rondinelli Roquetti, Liviam Elizabeth Cordeiro Beduschi, Amanda Salles Praia, Claudia Parucce Franco Okamoto, Vinicius Leite da Silva Carvalhaes, Evandro Albiach Branco, Bruna Barbezani, Emily Labandera, Kelsie Timpe, David Kaplan

**Affiliations:** 1 School of Forest Resources and Conservation, University of Florida, Gainesville, Florida 32611-0410, USA; 2 Institute of Energy and Environment, University of São Paulo, São Paulo CEP 05508-010, Brazil; 3 School of Arts, Sciences and Humanities, University of São Paulo, São Paulo CEP 03828-000, Brazil; 4 National Institute of Spatial Research (INPE), São José dos Campos CEP 12227-010, Brazil; 5 Department of Environmental Engineering Sciences, Engineering School of Sustainable Infrastructure and Environment, University of Florida, Gainesville, Florida 32611-0410, USA

**Keywords:** Hydrology, Sustainability, Interdisciplinary studies, Ecological epidemiology

## Abstract

Recognized as one of the world’s most vital natural and cultural resources, the Amazon faces a wide variety of threats from natural resource and infrastructure development. Within this context, rigorous scientific study of the region’s complex social-ecological system is critical to inform and direct decision-making toward more sustainable environmental and social outcomes. Given the Amazon’s tightly linked social and ecological components and the scope of potential development impacts, effective study of this system requires an easily accessible resource that provides a broad and reliable data baseline. This paper brings together multiple datasets from diverse disciplines (including human health, socio-economics, environment, hydrology, and energy) to provide investigators with a variety of baseline data to explore the multiple long-term effects of infrastructure development in the Brazilian Amazon.

## Background & Summary

Amazonia has long been the focus of worldwide attention for its remarkable biological and cultural diversity^1^ and for its role in regulating global biogeochemical and atmospheric cycles^2–4^. At the same time, the region has been increasingly viewed within recent decades as a frontier ripe with opportunities for economic growth and large-scale development^5^. These two, often conflicting views are closely intertwined, as national and international policies seek to advance Amazonian development^6^, and environmental interests struggle to conserve the region’s unique rainforest ecosystem^7,8^. Meanwhile, new growth policies increasingly focus on how to reconcile conservation and development^9,10^.

Within this context, science plays an essential role in documenting past successes and failures, thus enabling learning based on previous experience and new knowledge^11^. To facilitate evaluation of on-going infrastructure development in the Brazilian Amazon, researchers, policy makers and affected communities alike need reliable and comprehensive baseline data. Much of these data are scattered among different research institutions or available on difficult-to-access government websites. Furthermore, the information is often unpublished and/or recorded in different languages and formats. To overcome some of the difficulties involved with gathering such data, we compiled diverse datasets from the Brazilian Amazon, spanning public health, land use/land cover, climate, hydrology, hydroelectric dams, social development, economy, education, agriculture, and demography. Although these subjects come from disparate sources and were originally collected to address markedly different questions, taken together they begin to describe the complex social-ecological system that characterizes the Amazon and provide a platform from which to examine the multifaceted impacts of large-scale development in the region.

Our focus on the Amazon is timely, as large-scale infrastructure development projects driven by policies supporting energy production and economic growth threaten to disrupt the ecological dynamics of the region and transform local societies^12–16^. For example, the Initiative for the Integration of the Regional Infra-structure of South America (IIRSA), aims to transform Amazonia into a continental source of hydropower and an intermodal hub of roads, waterways, and railroads^17^. The Brazilian federal government’s Growth Acceleration Program (PAC) is also pushing transportation and energy development in Amazonia^18^, and oil and gas development are looming on the horizon^19^. While touted for bringing economic and social progress (e.g., electricity, transportation of goods and services, better health care, etc.), negative trade-offs often taint infrastructure development in the Amazon. For example, road construction and paving improves spatial mobility and access to goods and services for local populations but accelerates forest clearing and degradation^20,21^. These disturbances trigger biodiversity loss^22^ and other cascading effects that compromise ecosystem function^23^. Similarly, large hydroelectric dams in the Brazilian Amazon help meet increasing national demand for electricity but also create new mosquito habitat through expansion of surface water^24^ and reductions in forest cover, potentially increasing exposure to vector-borne diseases like malaria^25^. Furthermore, although promoted as ‘clean energy’, large dam reservoirs can produce substantial greenhouse gas emissions^26,27^. Dams also alter hydrological patterns, disrupting fisheries that human populations rely on for nutrition^28^. Increases in deforestation that inevitably accompany the establishment of infrastructure can also alter rainfall patterns^29,30^. In the case of hydropower plants, regional declines in rainfall and reductions in evapotranspiration provoked by forest loss reduce river discharge, compromising power generation itself^31^. Mega-development projects in the Amazon also spark a heavy influx of migrants that lead to rapid urban growth, prompting social and economic marginalization of significant portions of society within municipalities that are ill-equipped to support the needs of so many newcomers (e.g., sanitation, health care, electricity, schools)^5^.

This wide range of potential effects requires a broad data baseline to explore the complex implications of infrastructure development. A major strength of our database is that it presents a spectrum of social and biophysical variables together in one place^32^. Examples of interdisciplinary topics from our own work that require these type of data include the role of hydroelectric dams in social and economic development, impacts of infrastructure and deforestation on public health, and dam construction effects on hydrology and riverine communities (see Usage Notes for additional detail). While by no means comprehensive, our goal is to provide a database that will be continuously expanded upon and serve as a platform for ongoing efforts to explore the social-ecological impacts of infrastructure development in the Amazon from different perspectives.

## Methods

### Data collection

In this paper, we present multiple datasets for the Brazilian Amazon region. Our database comprises information from the nine states that fall within Brazil’s Legal Amazon, as defined by the Brazilian Institute of Geography and Statistics, IBGE (*Instituto Brasileiro de Geografia e Estatística*): Acre (AC), Amapá (AP), Amazonas (AM), Maranhão (MA), Mato Grosso (MT), Pará (PA), Rondônia (RO), Roraima (RR), and Tocantins (TO). Even though the Legal Amazon officially ends east of the 44th meridian, we include data for the entire state of Maranhão. To compile the database, we searched the web for indicators and drivers of environmental and social changes linked to infrastructure development projects in the region. We focused our search on variables related to socio-economic development, demography, land use and land cover, public health, hydrological systems, hydroelectric dams, and climate. Selected datasets are organized into five overarching themes described in detail below: Health, Environment, Socio-economics, Hydrology, and Hydroelectric Dams. All but the hydrology and hydroelectric dam datasets are aggregated at the municipal level.

### Health records

Public health records were drawn from Brazilian government websites. These records report the annual number of disease cases by municipality of residence, including dengue fever, cutaneous leishmaniasis, and HIV/AIDS. FIOCRUZ (*Fundação Oswaldo Cruz*) contributed additional edited data on malaria incidence, originally collected through the Brazilian government program, SIVEP-malaria (*Sistema de Informação de Vigilância Epidemiologica—Notificação de Casos*). Data files for dengue fever, cutaneous leishmaniasis, and HIV/AIDS cases were downloaded from the Brazilian Ministry of Health websites associated with SINAN (*Sistema de Informação de Agravos de Notificação*) and DATASUS (*Departamento de Informática do Sistema Único de Saúde*). Because links to these webpages are unstable, information regarding access to original datasets is detailed in Supplementary File 1.

### Environmental variables

Datasets associated with the Environment theme include monthly rainfall, original forest cover, and surface water cover. These variables were collected from three separate sources and summarized at the municipal level using ArcGIS (ArcMap 10.2), based on the 2010 Brazilian county map. Users interested in accessing original disaggregated environmental variables can access these data from the original sources indicated below.

1. *Rainfall* reports average monthly accumulated rainfall and length of dry season within the study region based on raster data from January 2000 to December 2010. Original raster data are available on a grid resolution of 0.25°×0.25° latitude/longitude, and were acquired from the National Aeronautics and Space Administration’s (NASA) Tropical Rainfall Measuring Mission (TRMM), product TRMM 3B43 (ref. 33). After isolating our study area and converting raster to point in ArcGIS, point values for monthly accumulated rainfall were averaged within municipalities. When no point fell within a given municipality, the rainfall point nearest to the municipality centroid was used. Next, municipal values for monthly accumulated rainfall were averaged over the 2000–2010 period. Length of dry season was calculated as the number of consecutive months with average monthly rainfall below 100 mm. This index has been extensively used to characterize drought in the region^34,35^. Original data can be accessed on-line at http://disc.gsfc.nasa.gov/datacollection/TRMM_3B43_V7.shtml.
2. *Original Forest Cover* displays total forested area (Km^2^) and percent forest cover per municipality estimated at the time Brazil’s ‘discovery’ by Europeans in 1500. These data are derived from a Brazilian vegetation map produced by IBGE (*Vegetação do Brasil* 1:500,000). Original maps display all vegetation types, but data presented here consider only forest vegetation classes. Using ArcMap 10.2, we subset the data to encompass the Legal Amazon states. We then used the Intersect tool to divide vegetation information by county and subsequently calculated forested area for each municipality. Total forest area corresponds to the aggregation of the following original vegetation classes: *Vegetação Ombrófila Aberta*, *Vegetação Ombrófila Aberta Aluvial*, *Vegetação Ombrófila Aberta Submontana*, *Vegetação Ombrófila Aberta Terras Baixas*, *Campinarana/Floresta Ombrófila*, *Floresta Ombrófila /Floresta Estacional*, *Floresta Estacional Decidual*, *Floresta Estacional Decidual Submontana*, *Floresta Estacional Semidecidual*, *Floresta Estacional Semidecidual Aluvial*, *Floresta Estacional Semidecidual Submontana*, *Floresta Estacional Semidecidual Terras Baixas*, *Floresta Ombrófila Densa*, *Floresta Ombrófila Densa Aluvial*, *Floresta Ombrófila Densa Montana*, *Floresta Ombrófila Densa Submontana*, *Floresta Ombrófila Densa Terras Baixas*. Original shape files of the vegetation map are downloadable at ftp://geoftp.ibge.gov.br/informacoes_ambientais/vegetacao/vetores/brasil/vegetacao/.
3. *Water Cover* is derived from the MODIS (Moderate Resolution Imaging Spectroradiometer) Water Mask, which can be downloaded from http://modis.gsfc.nasa.gov/data/dataprod/mod44w.php. This dataset relied primarily on data from the Shuttle Radar Topography Mission and was supplemented with MODIS 250 m data as necessary^36^ with data collected between 2000–2008. The spatial resolution of the dataset is 250 m. Using ArcGIS, the worldwide dataset was constrained to fresh water bodies within the Amazon study region, and polygons representing water areas were aggregated by municipality. From these data, total water cover area and percent cover was calculated for each municipality.

### Socio-economic indicators

An initial search was made for socio-economic data within two primary Brazilian sources: IBGE and the Institute for Applied Economic Research, IPEA (*Instituto de Pesquisa Econômica Aplicada*). Census data from IBGE are available at the municipal level and span variables related to demography, education, income, work, agriculture, quality of life, and poverty. Special consideration was given to the fact that Brazilian municipal boundaries have changed significantly over the past two decades, as new municipalities have been created and municipal borders shifted. These changes have been particularly prevalent in the Amazon region as new populations have migrated into the area. Between the 1991 and 2000 Brazilian censuses, 263 new municipalities were created within our study area (through the division of previously existing municipalities), and between 2000 and 2010, 15 more new municipalities were created (all within the state of Mato Grosso). These changes complicate comparisons over time. To avoid problems associated with these changes, we chose to use data from the Atlas of Human Development in Brazil (*Atlas do Desenvolvimento Humano no Brasil*—http://www.atlasbrasil.org.br), which summarizes a large subset of demographic census variables collected by IBGE in 1991, 2000, and 2010, and adjusts each variable to fit the municipal borders associated with the 2010 census. This was accomplished by using the original census tract level information from the 1991 and 2000 censuses and rearranging/recalculating the data to match 2010 municipal boundaries.

Within this dataset, we also include gold mining as an economic activity. Mining within Brazil is reported to the Brazilian National Department of Mines, DNPM (*Departamento Nacional de Produção Mineral*). Original shape files depicting all areas of reported mining activity in Brazil were downloaded by state from the DNPM website (http://sigmine.dnpm.gov.br/webmap/). For our dataset, we specifically selected the following gold mining categories, as defined by the DNPM: *lavra garimpeira* (small scale/alluvial mining), *licenciamento* (mining license), *requerimento de lavra* (mining authorization), *requerimento de licenciamento* (license authorization), and *concessão de lavra* (mining concession). Using ArcGIS, we matched the center of each mining polygon with the corresponding municipality and assigned gold mining presence/absence (1 and 0, respectively) to each municipality, based on the 2010 Brazilian county map.

### Amazon hydrology

Point data on water level (938 gauging stations), river discharge/flow (551 gauging stations), and daily precipitation (1342 gauging stations) were collected from the Brazilian Government National Water Agency, ANA (*Agência Nacional de Águas*) via their HidroWeb website (http://hidroweb.ana.gov.br/default.asp). Fluviometric gauging stations measure water level and/or river flow and pluviometric gauging stations record daily rainfall. Across our study region, including the Amazon, Tocantins/Araguaia, Paraná, and Atlantic watersheds, fluviometric and pluviometric stations have been measuring key hydrological variables on a daily basis since as early as 1922, although most data records range from 1965 to 2015. For water level, flow, and precipitation, we utilized the Hidro1.2 software package (widely used by ANA) to process the data and generate separate spreadsheet files for each gauging station.

### Hydroelectric energy

As an example of on-going infrastructure development, we present information on large hydroelectric dams in the Amazon. The dams dataset contains the following variables: dam name, affected river, construction start date, reservoir fill date, operation date, approved energy output, actual energy output, reservoir area, municipalities directly affected by the dam, and area of each municipality flooded by the dam. Only operating dams with capacity to produce over 30 MW of energy are included, following the Brazilian government’s criteria for ‘large’ dams (*Usinas Hidrelétricas de Energia*—UHE). Dam details were primarily acquired from the Brazilian National Agency for Electrical Energy, ANEEL (*Agência Nacional de Energia Elétrica*; http://www.aneel.gov.br). These data were extracted as .KMZ files (http://sigel.aneel.gov.br/kmz.html) and from subpages under *Compensação Financeira pela Utilização de Recursos Hídricos* (http://www.aneel.gov.br/aplicacoes/cmpf/gerencial/). Dates of dam construction and reservoir filling were gathered from various internet sources—usually from official websites associated with the dam or from government documents authorizing dam construction, but occasionally from newspaper or construction company websites. Whenever possible, reservoir fill dates were confirmed by comparing multiple Landsat satellite images in a time series following the construction date, using Google Earth Engine^37^.

### Known data anomalies

A large portion of the data found in our database (i.e., socio-economic variables) were originally collected by IBGE as part of the National Brazilian Censuses in 1991, 2000 and 2010. As mentioned above, however, municipal boundaries changed significantly between 1991 and 2010, with new municipalities created and others shrinking or expanding in size. This complexity makes comparisons across time periods difficult; however, thanks to a United Nations Development Programme (UNDP) initiative (in collaboration with IPEA and the *Fundação João Pinheiro*), the Atlas of Human Development in Brazil, the socio-economic data presented here have been adjusted to align with Brazil’s more recent 2010 municipal map. A more extensive data source covering an even wider range of socio-economic and development variables collected by the Brazilian Census is accessible through the IBGE website (http://www.ibge.gov.br); however, 1991 and 2000 data are not adjusted to match 2010 municipal boundaries, and therefore, downloadable municipal maps for each corresponding year—also available on the IBGE website—must be used to analyze the data, and comparisons across years are restricted. Also, certain variables within our Socio-Economics dataset were measured in 2000 and 2010, but not in 1991 (e.g., Gross Domestic Product [GDP]). In this case, we retained the variable in the 1991 dataset to maintain consistency across census years, but assigned missing values (NA) for all municipalities.

Although Health data in this paper did not undergo adjustments for municipal boundaries, all datasets report disease incidence from 2001 forward. After 2001, only three municipalities in the Amazon region were either newly created or underwent significant border changes. We designated values for these three municipalities from Mato Grosso (Ipiranga do Norte, Itanhaga, and Tapurah) as missing values (NA). Webpages originally accessed to download dengue fever, cutaneous leishmaniasis, and HIV/AIDS datasets are inconsistently available (i.e., links to these websites intermittently fail). We offer more details regarding web access to health datasets in Supplementary File 1.

Hydrology data also presented unique challenges in that gauging stations have recorded data over variable time spans (some date back 1922), and a substantial amount of data is missing or uncollected. Nonetheless, temporal overlap exists, and although patchy at times, these data provide the best available information to users interested in Amazonian hydrology and how it relates to the greater Amazon social-ecological system.

To facilitate the integration of hydrology data with other datasets, municipal code information was added to each fluviometric and pluviometric gauging station. During this process, we observed that some municipality names associated with gauging stations on HidroWeb did not match station locations when mapped on the 2010 municipality map. These stations likely retained the municipality name current at the time measurements started (e.g., the 1960s), creating a discrepancy where new municipalities have been established since monitoring began. Therefore, we confirmed, and when necessary updated, municipality names and municipal codes associated with hydrology stations, based on 2010 municipal boundaries.

## Data Records

The Amazon social-ecological database is available as comma-delimited text files (.csv) or as Microsoft Excel (.xlsx) files organized around the following themes: Health (incidence of malaria, dengue fever, cutaneous leishmaniasis, and HIV/AIDS), Environment (mean monthly rainfall and length of dry season, forest cover, and water cover), Socio-economics (including economy, social, education, agriculture, demography, and mining activity), Hydrology (river water level, river discharge/flow, and daily precipitation), and Hydroelectric Dams. All data are stored in the Dryad Digital Repository (Data Citation 1) and are also accessible through the Amazon Dams Network on-line data portal (Data Citation 2). A list of data sources used to build this database, along with data descriptions, spatial and temporal resolutions, and time spans is included in Table 1. The geographic scope of our study encompasses the Brazilian Amazonian states of Acre (AC), Amapá (AP), Amazonas (AM), Maranhão (MA), Mato Grosso (MT), Pará (PA), Rondônia (RO), Roraima (RR), and Tocantins (TO). All information is summarized at the municipality level (*n*=807), except for point-based hydrology data and hydroelectric dam variables. A summary of field names contained within each dataset is available for download along with the data (see ReadMe files). Covered time period varies by dataset (see Table 1).

**Figure 1:**
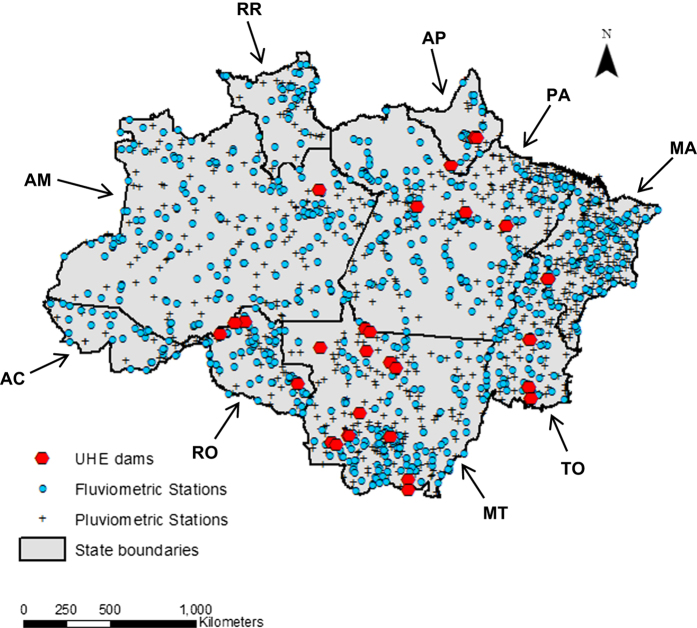
Map of the Legal Amazon, including states of Acre (AC), Amapá (AP), Amazonas (AM), Maranhão (MA), Mato Grosso (MT), Pará (PA), Rondônia (RO), Roraima (RR), and Tocantins (TO), showing locations of operating hydroelectric dams (>30 MW; *n*=22), and fluviometric and pluviometric gauging stations (*n*=947 and *n*=1342, respectively) that supply information to our social-ecological database. NOTE: most fluviometric stations overlap pluviometric stations.

Under the theme of HEALTH, cases of malaria, dengue fever, cutaneous leishmaniasis, and HIV/AIDS in the Brazilian Amazon are cataloged in four separate downloadable .csv files. We have also made available copies of the originally downloaded raw datasets, due to instability of links to on-line data sources. Malaria cases for *Plasmodium vivax* and for *Plasmodium falciparum* are reported monthly from 2003–2011. Cases of HIV/AIDS are reported annually from 2001 to 2014, and dengue fever and cutaneous leishmaniasis cases are reported annually from 2001–2012 and 2001–2013, respectively. We excluded pre-2000 data to avoid complications associated with changing municipal boundaries in the study region. For the ENVIRONMENT dataset, we captured average monthly rainfall and length of dry season (2000–2010), original forest cover, and water cover (2000–2008) in a single downloadable .csv file. Under SOCIO-ECONOMICS, three .csv files report on 99 socio-economic variables for Amazon municipalities in 1991, 2000, and 2010. These data span subjects of demography, education, income, work, agriculture, quality of life, poverty, and gold mining. The HYDROLOGY dataset contains a single Excel file for each fluviometric station reporting water level (*n*=938), each fluviometric station reporting river discharge/flow (*n*=551), and each pluviometric station reporting daily rainfall (*n*=1,342). Due to the large amount of data, hydrology station files are combined into a four zipped folders, according to watershed. Within each watershed folder, we also provide a summary table for fluviometric and pluviometric stations in .csv format that lists station code, station name, river, municipality code, municipality name, latitude, longitude, and data collection start and end dates for each gauging station. All operating HYDROELECTRIC DAMS in our study area that generate more than 30 MW (*n*=22) are included in the dams data file. Figure 1 displays all fluviometric and pluviometric stations included in our database, as well as locations of hydroelectric dams.

Across the entire database, missing values are identified as NA, except for hydrology datasets, where blank cells indicate missing data. To facilitate integration of datasets with Geographic Information Systems (GIS), we also provide downloadable shape files of the 2010 municipal Census map for the Brazilian Amazon.

## Technical Validation

Data on disease cases were drawn from the Brazilian government Ministry of Health website. While it is common for surveillance data to have several issues (e.g., under reporting), there is no clear way to correct for this, and we believe the data presented here represents the best available at this spatial scale. Socio-economic variables were reproduced from the Atlas of Human Development in Brazil, a reputable consortium of governmental and non-governmental entities, partially funded through UNDP. Similarly, raw data for Environment variables (rainfall, forest cover, and water cover) were downloaded from well-known, reliable on-line sources, including NASA and IBGE. To verify our calculations (up-scaling) of environment variables at the municipality level, we inspected data for abnormal values and nonsensical results.

Hydrology data was extracted from Brazil’s National Water Agency’s website, and we rely on the accuracy of the government’s data collection. Following data downloads, we double-checked data for errors and subsequently updated municipality names to agree with 2010 municipal boundaries, based on hydrology station coordinates. To the extent possible, using our surface water cover data, we also verified that fluviometric stations were located along waterways.

Data on currently operating hydroelectric dams were principally garnered from ANEEL, the Brazilian government agency responsible for authorizing and monitoring hydroelectric dams in Brazil. Operation dates are readily available through the ANEEL website. In addition, most large hydroelectric dams host a company website where they record technical information, such as reservoir size, energy output, and flooded municipalities. Within the ANEEL website, official legislation documenting different stages of dam approval provided additional details on operation timelines. Construction and filling dates were the most difficult to encounter, and in many cases this information was derived from company websites or newspaper reports. Whenever possible, dates for dam construction and reservoir filling were verified from multiple sources. If discrepancies arose, data were re-confirmed, and if any doubt remained, variables received an NA designation. Corrections to these dates are welcome and encouraged. As most of the information gathered came from Brazilian sources, Portuguese text and descriptions were translated into English and subsequently verified by a native Brazilian Portuguese speaker.

## Usage Notes

Many of the questions related to the development and conservation of the Amazon region require an interdisciplinary approach and thus, a broad historical and consistent database. Therefore, as much as possible, all data were adjusted to match the 2010 municipality map to provide an excellent resource for temporal comparisons and historical analysis across the different datasets. Data will be continuously updated and made available at http://www.amazondamsnetwork.org.

Because of the wide scope of our database, the information presented here can be used to answer a diverse set of research questions. Examples of highly interdisciplinary topics from our own work that require these type of data include:

1. Hydroelectric Dams and Development: Dams have historically been viewed as a means to facilitate and promote development. However, little empirical evidence stands behind this claim, particularly when development is broadly defined (e.g., including not only economic indicators but also indicators on health, education, etc.). We are currently evaluating whether large hydroelectric dams play a positive or negative role in development in Brazil using a broad set of municipal level development indicators;
2. Infrastructure, Deforestation and Health: Several tropical diseases are transmitted by vectors (e.g., snails, mosquitoes, flies, and fleas) that are influenced by changes in environmental factors, such as temperature, rainfall, vegetation and water bodies. We have been studying how deforestation and infrastructure development (roads, dams, mining) impact malaria by modifying the environment^38,39^;
3. Dam Construction and Hydrologic Change: Riverine communities in the Amazon region rely heavily on fish as their main protein source and the fertilization effect of flooding on their crops. We are currently investigating the hydrologic impacts of dam construction, how they differ by hydro-climatological region and dam type, and the likely impacts on Amazonian peoples’ livelihoods.

The topics listed above present examples of how a broad interdisciplinary database like ours can be used, but should not be interpreted as an exhaustive list of themes that could be addressed with these data. By making these data more widely (and easily) accessible, we envision they can be helpful in identifying and quantifying environmental, social, and economic pros/cons of development decisions and how they change over time. Ultimately, we expect that this work will promote a more evidence-based national and international discussion of development projects, motivating new growth policies that strive to reconcile conservation and development.

We invite users, researchers, and practitioners to add to our database and expand our efforts to improve the accessibility and scope of baseline data for the Amazon region. Depending on the research question and area of interest, other datasets not included this paper, such as road networks, soil maps, water quality data, and maps of conservation areas, indigenous and sustainable use reserves^40^, could supplement our Amazon database. Through collaboration and expansion of reliable, publicly available data, we strengthen our capacity to effectively research the impacts of infrastructure development and other interventions on the complex social-ecological system of the Amazon region.

## Additional Information

**How to cite this article:** Tucker Lima, J. M. *et al.* A social-ecological database to advance research on infrastructure development impacts in the Brazilian Amazon. *Sci. Data* 3:160071 doi: 10.1038/sdata.2016.71 (2016).

## Supplementary Material

Supplementary File 1

## Figures and Tables

**Table 1:** Description of datasets used to build the Amazon social-ecological database, organized under five themes: Health (HLT); Environment (ENV); Socio-economics (SOC); Hydrology (WAT); and Hydroelectric dams (DAM).

**Variable**	**Theme**	**Description**	**Spatial resolution**	**Temporal resolution**	**Time span**
Malaria*	HLT	Number of cases (*Plasmodium vivax* & *P. falciparum*)	County	Monthly	2003–2011
Dengue Fever†	HLT	Number of cases	County	Yearly	2001–2012
Cutaneous Leishmaniasis†	HLT	Number of cases	County	Yearly	2001–2013
HIV/AIDS‡	HLT	Number of cases	County	Yearly	2001–2014
Rainfall§	ENV	Mean monthly accumulated rainfall (mm) and average length of dry season	0.25°×0.25°/Averaged by County	Decadal average	2000–2010
Original Forest Cover||	ENV	Forest cover at the time of Brazil’s ‘discovery’	Map Scale: 1:5,000,000/ Aggregated by County	Pre-colonial Brazil	—
Surface Water Cover¶	ENV	Fresh water cover	250 m/Aggregated by County	2000–2008	2000–2008
Economy#	SOC	Economic variables related to GDP, poverty, income, and employment	County	Decadal	1991, 2000, 2010
Social#	SOC	Development variables including life expectancy, fecundity, mortality, access to water and electricity, and Human Development Index (HDI)	County	Decadal	1991, 2000, 2010
Education#	SOC	Literacy rates, school completion, school attendance rates, etc.	County	Decadal	1991, 2000, 2010
Demography#	SOC	Total population, by age class, male/female, urban/rural, etc.	County	Decadal	1991, 2000, 2010
Agriculture#	SOC	Cultivated crop area, agricultural production, and agricultural GDP	County	Decadal	1991, 2000, 2010
Gold Mining**	SOC	Presence (1) or Absence (0) of gold mining activity	County	Decadal	1991, 2000, 2010
River Water Level††	WAT	River level based on gauge measurements (cm)	Point	Daily	~1965–2015
River Flow††	WAT	Calculated river discharge/ flow based on station rating curves and measured level (m^3^s^−1^)	Point	Daily	~1965–2015
Precipitation††	WAT	Measured precipitation (mmperday)	Point	Daily	~1965–2015
Hydroelectric dams‡‡	DAM	Construction and operation details for hydroelectric dams energy production >30 MW; reservoir size, location, and energy output	Point (dam structure)	Date/Year	1967–2015

*Source: SIVEP-Malaria (Sistema de Informação de Vigilância Epidemiológica—Notificação de Casos).

†Source: SINAN (Sistema de Informação de Agravos de Notificação) (http://portalsinan.saude.gov.br).

‡Source: DATASUS (Departamento de Informática do Sistema Único de Saúde) (http://www.aids.gov.br).

§Source: TRMM (Tropical Rainfall Measuring Mission)/ NASA (http://trmm.gsfc.nasa.gov).

||Source: IBGE (Instituto Brasileiro de Geografia e Estatística) (http://www.ibge.gov.br).

¶Source: MODIS Water Mask -- Moderate Imaging Spectroradiometer (http://modis.gsfc.nasa.gov).

#Source: Human Development Atlas of Brazil (Atlas do Desenvolvimento Humano no Brasil) (www.atlasbrasil.org.br).

**Source: DNPM (Departamento Nacional de Produção Mineral) (http://sigmine.dnpm.gov.br).

††Source: ANA (Agência Nacional de Águas) (http://hidroweb.ana.gov.br).

‡‡Source: ANEEL (Agência Nacional de Energia Elétrica) (http://www.aneel.gov.br).
