# Supplementary material for: A social-ecological database to advance research on infrastructure development impacts in the Brazilian Amazon
Source: Sci Data. 2016 Aug 30;3:160071. doi: 10.1038/sdata.2016.71 (PMC5004584; doi:10.1038/sdata.2016.71)
Supplement: Supplementary File 1 [file sdata201671-s1.doc]

**Supplementary File 1.** Additional remarks and details regarding unstable links to health datasets.

We originally downloaded dengue fever (DF) and cutaneous leishmaniasis (CT) case data from the Brazilian Ministry of Health’s SINAN (*Sistema de Informação de Agravos de Notificação*)/DATASUS (*Departamento de Informática do Sistema Único de Saúde*) website at http://dtr2004.saude.gov.br/sinanweb/index.php, and HIV/AIDS data were obtained from the TABNET (*Informações de Saúde*) resource associated with the Brazilian government’s HIV/AIDS website (<http://www2.aids.gov.br/final/dados/dados_aids.asp>). Unfortunately, since the original download of these datasets, links to these websites have been unstable and data access is inconsistent.

Therefore, we present alternatives to the above links, where we surmise that the data will be available in the near future. For DF, the webpage <http://portalsinan.saude.gov.br/dengue> includes a link to case records (*Tabulação de dados*) for this disease under the subtitle, “Dados epidemiológicos e estatísticas”, although the link was inactive at the time this paper was published and could not be confirmed. Likewise for CT, the webpage <http://portalsinan.saude.gov.br/leishmaniose-tegumentar-americana> includes a non-functioning link to CT case records (*Tabulação de dados*). Furthermore, access to HIV/AIDS case data is linked through the website <http://www.aids.gov.br/pagina/tabulacao-de-dados> although, as with DF and CT, the link (A*cessar o sistema*) was inactive at the time of publication.

We sincerely hope that the functionality of the original health data links will return, but in order to ensure that other researchers can access these data in the future, copies of the original downloaded datasets (prior to data manipulation) are available in the Dryad Data Repository (“Original Health Datasets”).
